# Neutrophil to Lymphocyte Ratio as a Biomarker for Predicting the Coronary Artery Abnormality in Kawasaki Disease: A Meta-Analysis

**DOI:** 10.1155/2022/6421543

**Published:** 2022-10-11

**Authors:** Shirin Sarejloo, Matin Moallem Shahri, Pouria Azami, Alec Clark, Ethan Hass, Maryam Salimi, Brandon Lucke-Wold, Shahram Sadeghvand, Shokoufeh Khanzadeh

**Affiliations:** ^1^Cardiovascular Research Center, Shiraz University of Medical Sciences, Shiraz, Iran; ^2^Department of Thoracic Surgery, Thoracic Surgery Research Center, School of Medicine, Mashhad University of Medical Sciences, Mashhad, Iran; ^3^University of Central Florida College of Medicine, USA; ^4^Bone and Joint Diseases Research Center, Shiraz University of Medical Sciences, Shiraz, Iran; ^5^Department of Neurosurgery, University of Florida, Gainesville, Florida, USA; ^6^Pediatric Health Research Center, Tabriz University of Medical Sciences, Tabriz, Iran; ^7^Student Research Committee, Tabriz University of Medical Sciences, Tabriz, Iran

## Abstract

We conducted a systematic review and meta-analysis on the relationship between the neutrophil to lymphocyte ratio (NLR) and coronary artery abnormalities (CAA) in patients with Kawasaki disease (KD), according to the Preferred Reporting Items for Systematic Reviews and Meta-Analyses (PRISMA) statements. We searched PubMed, Scopus, Web of Science, Embase, TRIP, Google Scholar, and ProQuest up to the 8th of August 2022. This was done to retrieve eligible studies. No date or language limitations were considered in this study. Methodology quality assessment was conducted according to the Newcastle–Ottawa scale (NOS). Standard mean difference (SMD) and its 95% confidence interval (CI) were used to depict the pooled continuous variables. Finally, 17 articles with 6334 KD patients, of whom 1328 developed CAA, were enrolled in this meta-analysis. NLR level was significantly higher in KD patients with CAA compared to those without CAA (SMD =0.81; 95% CI =0.05–1.57, *P* = 0.03). In addition, NLR level was significantly higher in patients with coronary artery aneurysms than those without coronary artery aneurysms (SMD =2.29; 95% CI =0.18–4.41, *P* = 0.03). However, no significant association between NLR and coronary artery dilation was observed in this meta-analysis (SMD =0.56; 95% CI = -0.86–1.99). There was no publication bias for the pooled SMD of NLR for coronary artery abnormality in KD (Egger's test *P* = 0.82; Begg's test *P* = 0.32). The NLR may be useful in monitoring CAA development in these patients and may further imply a mechanistic role in potential inflammation that mediates this process.

## 1. Introduction

Kawasaki disease (KD) is an acute, febrile vasculitis that occurs predominantly in children under 5 years old. It presents with a classic presentation that may include erythema of the palms and soles, maculopapular rash, conjunctival injection, oral mucosal abnormalities, and cervical lymphadenopathy [1]. Coronary artery abnormalities (CAA) have been identified in a subset of KD patients. One of the major complications is dilation of one or more arteries. This varies from minor arteries to large (or giant) CA aneurysms [1]. While dilations of the CAs have been found to regress in most cases, large aneurysms have demonstrated a tendency to persist for years, predisposing to rupture [2]. Further, identification of large aneurysms in childhood has been associated with a significantly increased risk of developing adverse cardiac events in adulthood, including unstable angina pectoris and myocardial infarction [3]. The prevalence of large CA aneurysms in KD has diminished in recent decades, with studies reporting 0.1-0.5% incidence among KD cases since the introduction of intravenous gamma globulin (IVGG) treatment [4, 5]. Despite this downward trend, it is estimated that 5% of myocardial infarction cases in adults under 40 years old in the United States are caused as a consequence of the sequelae of KD [6].

No one etiology has been identified for KD or related CAA, although genetic and environmental factors have been speculated [1]. Elevations of proinflammatory blood markers during the acute phase of KD [7] suggest that a dysregulation of the inflammatory response is involved in the disease progression. The degree of this dysregulation may also determine the risk of developing CA aneurysms, as arterial wall infiltration by immune cells like neutrophils is an early pathological finding of KD-related CAA. This is followed by predominantly lymphocytic infiltrates in the subacute phase [8, 9].

Neutrophil to lymphocyte ratio (NLR) analysis of the peripheral blood is becoming a popular, cost-effective tool for the clinical assessment of inflammatory involvement in a wide variety of diseases [10, 11]. Neutrophils mediate nonspecific inflammation by combating pathogens and releasing proinflammatory cytokines when recruited to the tissues. When neutrophilic recruitment becomes abnormally high, leukocytosis can be identified, as in KD [12]. Conversely, lymphocytes carry out functions of adaptive immunity by targeting the response rather than promoting nonspecific inflammation. There is growing evidence that both innate and adaptive immune cells mediate the systemic and tissue-specific effects of KD-like CA aneurysms [9]. In light of this, several studies have suggested that NLR calculation may aid in the clinical assessment of the inflammatory aspect of KD, serving as a metric for risk stratification and prediction for adverse cardiac events in adulthood [13–26].

Herein, we conducted a systematic review and meta-analysis on the relationship between NLR and CAA in KD patients. To the best of our knowledge, this is the first systematic review and meta-analysis on this topic.

## 2. Method

This systematic review was performed according to the Preferred Reporting Items for Systematic Reviews and Meta-Analyses (PRISMA) statements. Two investigators conducted study selection, data extraction, and quality assessment steps separately.

### 2.1. Study Selection

We searched PubMed, Scopus, Web of Science, Cochrane library, ScienceDirect, Embase, ProQuest, TRIP, and the Google Scholar up to the 8th August 2022 to retrieve eligible studies. The literature search was performed using the following keywords: “neutrophil to lymphocyte ratio,” “neutrophil-to-lymphocyte ratio,” or “NLR” in combination with “coronary” and “Kawasaki.” The exact search strategy of each database is shown as supplementary file A. Moreover, the reference list of the relevant articles was also screened to determine additional studies. No date or language limitations were considered in this study.

### 2.2. Inclusion Criteria

The inclusion criteria were as follows: (i) case-control or cross-sectional design; (ii) peer-reviewed full-text publications; (iii) reporting the blood NLR data as mean ± SD or median [range or interquartile range]; (iv) comparing KD patients with and without CAA including those with aneurysms and dilation.

### 2.3. Exclusion Criteria

The exclusion criteria were as follows: (i) data were unavailable or only reported in abstracts; (ii) articles were submitted by the same authors or institution which might have overlapping patients; (iii) duplicate publications; (iv) reviews, editorials, meeting abstracts, case reports, and non-comparative studies.

### 2.4. Data Extraction

The extracted information included the name of the first author, publication year, study design, country, number of patients in case and control groups, and the values of NLR in each group.

### 2.5. Methodology Quality Assessment

Methodologic quality assessment was conducted according to the Newcastle–Ottawa scale (NOS) with a score range of 0–9 points, and high quality was defined as a score of ≥6.

### 2.6. Statistical Analysis

The statistical analysis was performed by using STATA version 12.0 (Stata Corporation, College Station, TX, USA). Standard mean difference (SMD) and its 95% confidence interval (CI) were used to depict the pooled continuous variables. In addition, the Cochrane *Q* test (*χ*^2^) and *I*^2^ test were used to evaluate statistical heterogeneity among the studies; if significant heterogeneity existed among included studies (*P* < 0.05 and *I*^2^ ≥ 50%), a random-effect model was used; otherwise, the fixed-effect model was conducted. The publication bias was assessed using the funnel plot, Egger test, and Begg test. A two-tailed *P* < 0.05 was considered statistically significant.

## 3. Results

### 3.1. Study Selection

**Figure 1:**
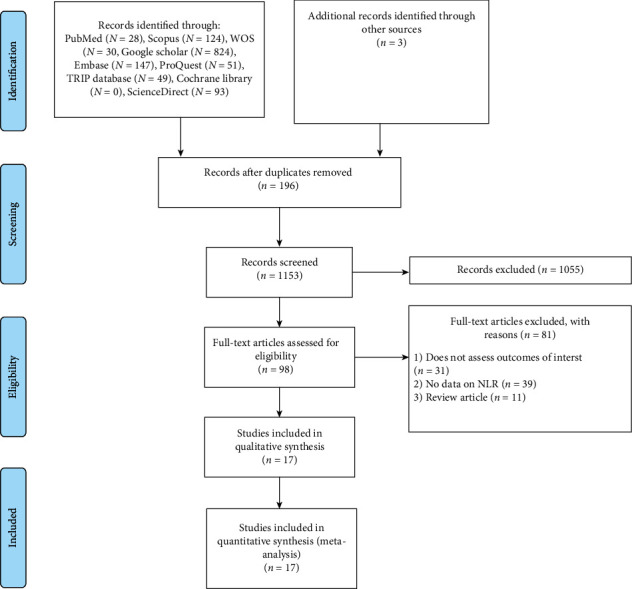
Flow chart of search and study selection.

The flow chart diagram of the study selection is shown in Figure 1. A total of 1349 articles were yielded through a primary study search. Of these studies, 28 were from PubMed, 124 from Scopus, 30 from WOS, 824 from Google Scholar, 147 from Embase, 51 from ProQuest, 49 from TRIP database, 93 from ScienceDirect, and three from other sources. Among the studies, 196 articles were removed because of duplication, and 1055 publications were excluded due to reviews, meeting abstracts, no reporting on NLR data, and not being pertinent to CAA after screening the title and abstract. Thus, the remaining 98 articles were left for the full-text review. Of the 98 studies, 11 articles were removed because they were reviews, 39 were removed due to the lack of available data on NLR, and 31 were excluded because they did not assess outcomes of interest.

Finally, 17 articles with 6334 KD patients, of whom 1328 developed CAA, were enrolled in this meta-analysis [13–29]. The list of included studies is shown in supplementary file B.

### 3.2. Characteristics of Included Studies

All the selected studies were published from 2015 to 2021. Of 17 articles, seven came from China [21, 22, 24, 26–29], four from Korea [16, 19, 20, 25], three from Turkey [13, 17, 18], one from Japan [23], one from Taiwan [14], and one from Thailand [15]. Sixteen studies were retrospective [13–23, 25–29], and one study was prospective [24]. Twelve studies were written in English [13–17, 19–25], one in Turkish [18], and two studies in Chinese [26, 29]. Sixteen studies were published as journal articles [13–21, 23–29] and one study as a preprint [21]. The basic characteristics and data of interest in the eligible studies are summarized in Table 1. The demographic data of samples included in the meta-analysis is shown in Table 2. Moreover, the methodological quality of the included articles was assessed according to NOS, and the scores ranged from 5 to 8. This indicates that the quality of selected studies was moderate to high.

### 3.3. The Association of NLR with Coronary Artery Abnormality in Kawasaki Disease

**Figure 2:**
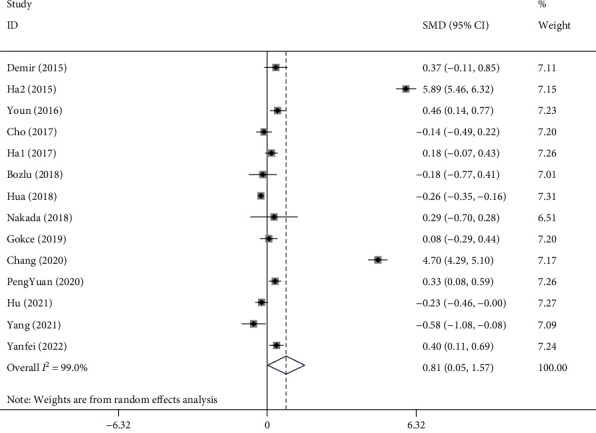
Meta-analysis of differences in NLR level between Kawasaki patients with coronary artery abnormality and those without coronary artery abnormality.

A total of 14 studies [13, 14, 16–26, 29] involving 5952 KD patients, of whom 1218 developed CAA, were included in the meta-analysis. Random-effect model was applied since significant heterogeneity was present (*I*^2^ = 99.0%; *P* < 0.001). From the results of our meta-analysis, NLR level was significantly higher in KD patients with CAA compared to those without CAA (SMD =0.81; 95% CI =0.05–1.57, *P* = 0.03, Figure 2).

### 3.4. The Association of NLR with Coronary Artery Aneurysm in Kawasaki Disease

**Figure 3:**
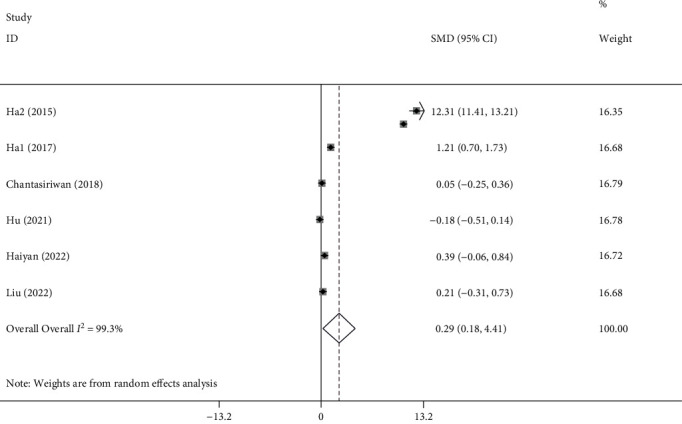
Meta-analysis of differences in NLR level between Kawasaki patients with coronary artery aneurysm and those with normal coronary arteries.

A total of six studies [15, 19–21, 27, 28] with 1916 KD patients, of whom 180 developed coronary artery aneurysm, were included in this meta-analysis. The heterogeneity tests showed the extinction of significant heterogeneity (*I*^2^ = 99.3%; *P* < 0.001), so the random-effect model was used. The results suggested that NLR level was significantly higher in patients with coronary artery aneurysms (SMD =2.29; 95% CI =0.18–4.41, *P* = 0.03, Figure 3) than in those without coronary artery aneurysms.

### 3.5. The Association of NLR with Coronary Artery Dilation in Kawasaki Disease

**Figure 4:**
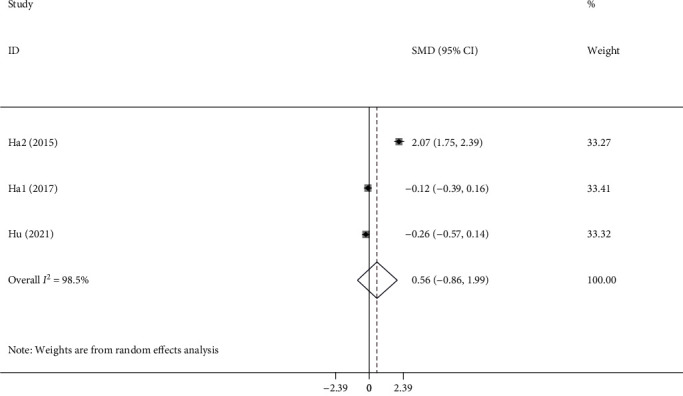
Meta-analysis of differences in NLR level between Kawasaki patients with coronary artery dilation and those with normal coronary artery.

Three articles with 1612 KD patients [19–21], of whom 148 developed coronary artery dilation, were also included for the meta-analysis regarding the association between NLR and coronary artery dilation in KD. The random-effect model was used because of the extinction of significant heterogeneity in the heterogeneity tests (*I*^2^ = 98.5%; *P* < 0.001). However, no significant association between NLR and coronary artery dilation was observed in this meta-analysis (SMD =0.56; 95% CI = -0.86–1.99, Figure 4).

### 3.6. Publication Bias

**Figure 5:**
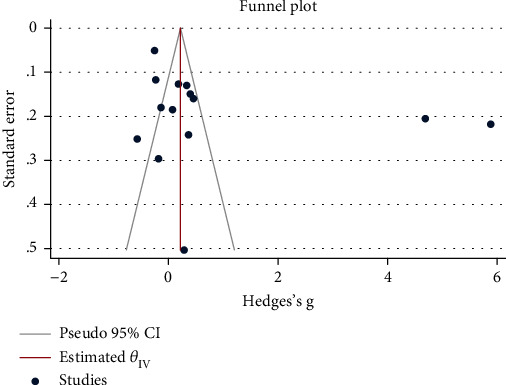
Funnel plot assessing publication bias.

The assessment of publication bias of the included studies was performed using Begg's and Egger's tests. From the results of the publication bias test, we found that there was no evidence of publication bias for the pooled SMD of NLR for coronary artery abnormality in KD (Egger's test, *P* = 0.82; Begg's test, *P* = 0.32, Figure 5).

## 4. Discussion

**Figure 6:**
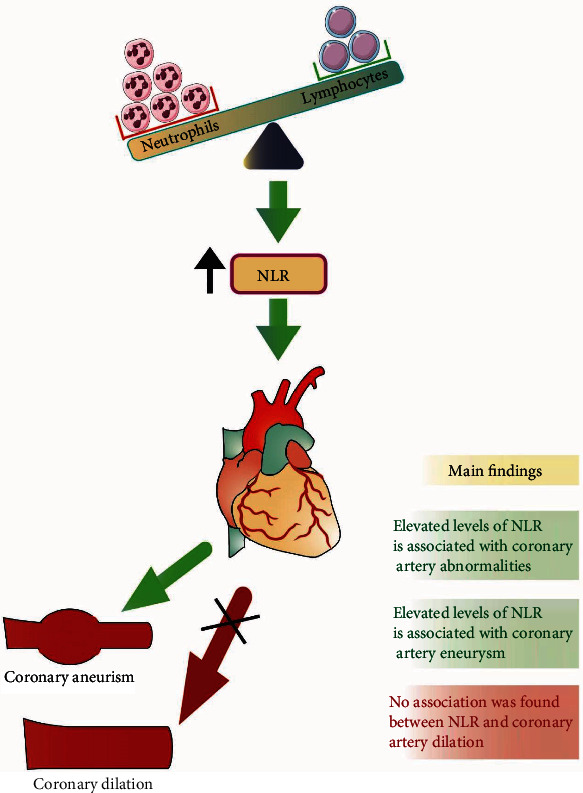
Main findings of the study.

The outcome of this study demonstrates that KD patients with CAA had significantly elevated levels of NLR compared to those without CAA. Statistically significant elevations in NLR were also demonstrated in patients with coronary artery aneurysms compared with those without CAA. No significant elevations in NLR were demonstrated in those with coronary artery dilation compared to those without CAA and those with coronary artery aneurysms (Figure 6). Thus, results from this study suggest that the NLR may be useful in monitoring CAA development in these patients and may further imply a mechanistic role in inflammation that mediates this process.

### 4.1. Prognostic Value of NLR Related to Long-Term Sequelae of KD

The most severe and frequent complication of KD is the development of coronary artery involvement. The recent introduction of treatment with IVIG has reduced this problem. Despite treatment with intravenous gamma globulin, 2% to 4% of patients have coronary abnormalities or develop coronary aneurysms [30]. Those with giant aneurysms are at risk for stenosis and myocardial ischemia/infarction, which requires systemic anticoagulation with frequent follow-up. This predisposes to frequent stress testing and coronary angiography. In rare cases, patients will have coronary artery bypass grafting. Those with less severe coronary involvement need antiplatelet therapy and infrequent noninvasive testing [31]. Patients with normal echos after the acute phase are not treated, but the future impact of the disease is not certain, particularly in adult-onset coronary artery disease [32]. NLR might be associated with CAA and might predict long-term cardiovascular risk in KD patients, so it can help clinicians in the risk stratification of these patients. This aids in the development of diagnostic and therapeutic modalities to prevent mortality and morbidity in such patients.

### 4.2. NLR and Inflammation

It is well-known that neutrophils play a critical role in the incitement of the proinflammatory response in the body, including both acute and chronic inflammatory states [12, 33, 34]. Furthermore, a reduction in lymphocyte count is commonly seen during proinflammatory states due to the modulatory role that lymphocytes play in these states [35]. Thus, the inverse relationship between neutrophils and lymphocytes makes the NLR a potentially optimal marker in proinflammatory states or conditions, such as CAA in KD patients.

As it pertains to the development of CAA in KD patients, a proposed mechanism of action that has been backed by significant evidence is the direct increased inflammatory response leading to arterial dilation and subsequent aneurysm formation. Several inflammatory cytokines have now been linked to this phenomenon, including IL-6, IL-8, TNF-*α*, IFN-*γ*, and C-reactive protein (CRP) [36–39]. Research has demonstrated that neutrophils routinely respond to several signaling pathways on the biochemical level, ultimately leading to the direct production of several of these cytokines [40, 41]. These cytokines manifest the inflammatory state by several mechanisms, including endothelial dysfunction and collagen destruction. This leads to the compromised structural integrity of coronary arteries, which directly contributes to intimal thickening and compromised laminar blood flow, eventual thrombosis, or aneurysm development [42]. Thus, neutrophils may be involved in both the initial incitement of and propagation of inflammation in the setting of disease progression. In either case, the NLR appears to be a prime candidate for a reliable inflammatory marker in these conditions.

### 4.3. NLR and Arterial Abnormalities

Potential mechanisms by which the NLR can incite CAA development in KD patients can be divided into neutrophil-derived and lymphocyte-derived etiologies.

Neutrophils have been, and have largely remained, the main mediator of the development of CAA seen in KD patients [43]. Researchers suspect that neutrophils begin to infiltrate the coronary arteries at approximately 1-2 weeks after the development of KD, which are then replaced by monocytes. With the eventual resolution of inflammation after approximately two months after fever onset, aneurysm formation occurs [43]. Several studies have highlighted the significant increase in neutrophil count seen in these patients that distinguish it from other viral etiologies [44, 45]. In more recent years, the role of neutrophils in the development of CAA in KD patients has been further characterized. Necrotizing arteritis has been described as a neutrophil-mediated process characterized by the gradual destruction of the adventitia of the endothelium. Both the onset and completion occur within the first two weeks consistent with fever period [46]. Neutrophil extracellular traps (NETs) are a mechanism specific to neutrophils. It is characterized by morphological changes that facilitate the trapping of pathogens by both oxidative and non-oxidative mechanisms [47]. NETs have now been demonstrated to play a role in inflammation in several autoimmune diseases, such as rheumatoid arthritis, systemic lupus erythematosus (SLE), and anti-neutrophilic cytoplasmic antibody- (ANCA-) associated vasculitis [48]. Even more recently, evidence of NET production in preclinical models mimicking KD conditions and clinical models exposed to KD serum has been demonstrated [49, 50]. Vascular endothelial growth factor (VEGF) production has also been hypothesized as a mechanism by which CAA develops, with the early production of VEGF in the acute phase seen predominately by neutrophils. In the chronic phase, production is seen predominately by mononuclear cells [51]. The exact etiology of this relationship is unclear but likely due, at least in part, to vascular injury and remodeling [51]. Furthermore, increased neutrophil respiratory burst, a marker of neutrophil activation, has been demonstrated in KD patients with CAA compared to healthy controls or KD patients without CAA [52]. This adds credence to the assertion that neutrophils are not only produced in increased quantities in this context but likely play a more active role in the pathogenesis of CAA.

Evidence for the mechanistic role of neutrophils in CAA development is most convincing in the acute phase of KD (1-2 weeks). Indeed, it has been shown that neutrophils infiltrate coronary arteries in KD most rapidly in the early stage of the disease, with peaks in macrophage and lymphocytes later in the disease course [53]. However, in the subacute and chronic phases, evidence for the role of other cells, particularly lymphocytes, is amassing [42]. A decrease in lymphocyte count is typically seen with systemic inflammatory responses [35]. Thus, as a condition where systemic inflammatory responses are seen, KD would logically be expected to manifest with a decreased lymphocyte count. However, studies in the context of CAA in KD tend to demonstrate an increase in lymphocyte response, particularly T-lymphocyte activation. This remains the main mediator of CAA in the post-acute phase of KD [54–56]. A concurrent type I interferon response has also been characterized alongside this T-lymphocyte activation, which some have speculated as potentially due to a presently unidentified infectious (i.e., viral) cause [54]. Interestingly, recent studies have also suggested a role of lymphocytes and even macrophages in the pathogenesis of CAA in the acute phase of the disease, with demonstrated transmural infiltration of primarily CD8 T lymphocytes and macrophages mainly demonstrated in the adventitial layer [57].

Although this has been purported to be a potential cause, a viral or bacterial etiology for KD has yet to be identified. Given that these findings seem to conflict on a superficial level with the logical assumption that lymphopenia, rather than lymphocytosis, would be seen in KD, a further explanation in the context of the results of this study is warranted. Firstly, the results of the aforementioned study mention that the evidence for the T-lymphocyte activation in KD is most significant in subacute or chronic KD cases [54]. If this is the case, then the lymphopenia most associated with systemic inflammatory responses would likely predominate in the acute phase of KD. Since lymphopenia would further increase the NLR in the setting of acute disease, and the classic clinical presentation is well-known and easily identifiable in the clinical setting, this would strengthen the case for using NLR as a screening marker for CAA in KD patients upon the patient's initial presentation. Moreover, it is also possible that even if lymphocytosis is present in these patients, the relative increase in neutrophil count may be much larger than the marginal increase in lymphocyte count, thus elevating the NLR regardless.

### 4.4. Biomarker Usage and Pharmacologic Insights

The utility of the NLR as a predictive biomarker for the development of CAA has been demonstrated in recent years, with one study reporting an NLR>3.5 as an independent risk factor for developing CAA in KD patients [14]. One study also showed that an IL-10 level greater than 8 pg/displayed a sensitivity of 75% and specificity of 64.4% when predicting the concurrent development of coronary artery lesions before intravenous immunoglobin (IVIG) administration [37]. After IVIG administration, an Il-6 level greater than 10 pg/mL had a sensitivity of 67.9% and a specificity of 81.7% for predicting the presence of coronary artery lesions, and an IL-10 level greater than 6 pg/mL had a sensitivity of 53.6% and specificity of 86% for predicting the presence of coronary artery lesions [37].

Research has also demonstrated that IVIG given early in the course of disease showed a quicker reduction in circulating neutrophils and a decrease in coronary artery lesion formation compared to patients who received aspirin only or those who received IVIG later in the disease course [14]. This not only demonstrates the potential efficacy of IVIG in reducing the CAA sequelae but also implies a mediating relationship between increased neutrophil count and CAA development. Moreover, IVIG administration can decrease several cytokines, including IL-6, IL-10, TNF-*α*, and IFN-*γ*, in patients with KD [37, 38]. In patients with concurrent CAA, the response of certain cytokine levels to IVIG may vary, with one study showing that levels of TNF-*α* may slightly increase in KD patients with coronary artery lesions compared to greatly decreased levels seen in KD patients without coronary artery lesions [37]. Furthermore, a recent meta-analysis demonstrated the potential diagnostic utility of the NLR as an independent predictor for IVIG-resistant KD. Namely, the NLR was shown to have a sensitivity of 66% and a specificity of 71% for predicting IVIG resistance in these patients [58]. These findings increase the credibility of the NLR in overall context for KD, demonstrating that it can serve as a useful tool in predicting response to first-line treatment as well as in predicting outcomes.

Given the results of these studies, the role that the NLR may play in diagnosing CAA in KD patients is promising. It can be used to develop medications aimed at reducing neutrophil count and activation and prevent long-term complications.

### 4.5. Limitation

The findings of this report are subject to some limitations. First, heterogeneity in studies was greater than expected due to various treatment regimens, duration of recorded stays, center protocols, different study populations, timing of blood tests from which NLR was calculated, and different study designs. Second, several of the studies are limited by bias, whether selection or publication, which should be considered. Third, the effect size for several of the tests was limited to a few studies. Thereby widespread adoption and applicability are again a concern warranting further investigation.

## 5. Conclusion

In conclusion, results from our study demonstrate that patients with KD and concurrent CAA had elevated levels of NLR compared to KD patients without CAA. NLR represents a unique inflammatory marker whose elevation in CAA implicates immune system imbalance in the pathogenesis of the disease. Further, our findings support NLR to be a promising biomarker that can be readily integrated into clinical settings to aid in the prediction and prevention of CAA. Given the implications for its role in inflammation, the NLR may be a useful tool for early monitoring in these patients. Evidence suggests that it may be most useful in the acute phase of the disease. This evidence also warrants investigation into new drug targets for developing novel medications. With the development of new biomarkers and therapeutic modalities, we can better prevent and treat CAA in KD with an emphasis on decreasing long-term morbidity and mortality.

## Figures and Tables

**Table 1:** General characteristic of included studies.

Author	Year	Country	Design	CAA group		Non-CAA group						NOS score
						Total		CA aneurysm		CA dilation		
				N	NLR	N	NLR	N	NLR	N	NLR	
Demir	2015	Turkey	R	49	1.50±1.28	26	2.02±1.63	_	_	_	_	5
Ha2	2015	Korea	R	525	1.20±0.07	62	2.21±0.49	15	4.72±1.72	47	1.39±0.22	7
Youn	2016	Korea	R	168	1.71±1.40	52	2.72±3.79	_	_	_	_	8
Cho	2017	Korea	R	158	4.78±4.88	38	4.15±3.50	_	_	_	_	7
Ha1	2017	Korea	R	613	3.27±2.48	69	3.75±3.81	15	6.53±7.37	54	2.98±2.83	6
Bozlu	2018	Turkey	R	43	3.59±3.41	15	2.99±2.83	_	_	_	_	5
Chantasiriwan	2018	Thailand	R	162	_	_	4.16±7.18	55	4.56±8.52	_	_	6
Hua	2018	China	R	1606	2.60±2.30	523	2.03±2.00	_	_	_	_	8
Nakada	2018	Japan	R	197	4.05±8.42	4	6.52±12.71	_	_	_	_	7
Gokce	2019	Turkey	R	114	2.17±1.14	39	2.29±2.51	_	_	_	_	6
Chang	2020	Taiwan	R	238	2.73±0.15	127	3.95±0.39	_	_	_	_	6
PengYoun	2020	China	R	513	2.63±2.28	67	3.40±2.50	_	_	_	_	7
Hu	2021	China	R	326	2.22±2.28	94	1.72±1.66	40	1.81±1.69	47	1.64±1.63	7
Yang	2021	China	P	28	4.20±3.80	38	2.40±2.50	_	_	_	_	6
Haiyan	2022	China	R	_	_	41	1.30± 1.02	37	1.93± 2.09	_	_	7
Liu	2022	China	R	_	_	69	5.52± 4.52	18	6.42± 3.67	_	_	7
Yanfei	2022	China	R	64	4.5± 5.1	156	3.1± 2.6	_	_	_	_	6

N: number; NLR: neutrophil to lymphocyte ratio; R: retrospective; P: prospective; CAA: coronary artery abnormality.

**Table 2:** Demographic data of samples included in the meta-analysis.

Author	Sample size	CAA group		Non-CAA group		Total sample	
		Age ^∗^	Male percentage	Age^∗^	Male percentage	Age^∗^	Male percentage
Demir	75	31± 44	73%	36 ±44	55%	34 ±46	61%
Ha2	587	27.73± 3.19	82.3%	34.32± 0.99	55%	_	_
Youn	220	32.56± 29.40	69%	24.91± 19.47	61%	_	63%
Cho	196	_	_	_	_	32± 21	59%
Ha1	682	_	_	_	_	30.0 (14.0-46.0)	57.9%
Bozlu	58	_	_	_	_	52.56±22.99	60%
Chantasiriwan	217	14[3-168]	60%	18[2-79]	64%	_	_
Hua	2129	19 [9–33]	69%	23 [11–47]	59%	_	_
Nakada	201	39.5 [20–62]	75%	24 [2–159]	50%	24 [2-159]	51%
Gokce	153	28 [15-46]	69%	27 [15-47]	64%	_	_
Chang	365	1.4[0.7-2.4]	72%	1.5[0.8-2.6]	54%	_	_
PengYoun	580	34.4± 30.1	70%	28.4± 22.5	65%	_	_
Hu	420	_	70%	_	59%	2.4 years[2 months-11 years]	62%
Yang	66	2.1 ± 1.3	66%	2.5 ± 1.5	61%	_	_
Haiyan	78	2.4 (1.8-3.7)	51.4%	3.0 (2.0-4.9)	65.9%	_	_
Liu	87	35.44 ± 36.71	78%	36.77 ± 22.54	75%	36.49 ± 25.86	76%
Yanfei	220	3 ± 2	69	2.9 ± 2.1	58	_	_

SD: standard deviation; CAA: coronary artery abnormality. ^∗^Expressed as mean ± SD or median (IQR) or median [range]. All ages are expressed as months, except for Haiyan and Chang.

## Data Availability

All data generated or analyzed during this study are included in this published article.

## Supplementary Materials

Supplementary Materials The exact search strategy of each database is shown as supplementary file A. The list of included studies is shown in supplementary file B.
