# Supplementary material for: Neutrophil to Lymphocyte Ratio as a Biomarker for Predicting the Coronary Artery Abnormality in Kawasaki Disease: A Meta-Analysis
Source: Dis Markers. 2022 Oct 11;2022:6421543. doi: 10.1155/2022/6421543 (PMC9578863; doi:10.1155/2022/6421543)
Supplement: Supplementary Materials — The exact search strategy of each database is shown as supplementary file A. The list of included studies is shown in supplementary file B. [file 6421543.f1.zip › Supplementary file A.docx]

PubMed

N=28

(("neutrophil"[All Fields] AND "lymphocyte"[All Fields] AND "ratio"[All Fields]) OR "neutrophil-to-lymphocyte"[All Fields] OR "NLR"[All Fields]) AND "coronary"[Title/Abstract] AND "Kawasaki"[Title/Abstract]

Scopus

N=124

( ( ALL ( ( neutrophil  AND  lymphocyte  AND  ratio )  OR  ( neutrophil-to-lymphocyte )  OR  NLR ) ) )  AND  ( ALL( coronary ) AND TITLE-ABS-KEY  (Kawasaki)  )

WOS

N= 30

#1

All=((neutrophil  AND  lymphocyte  AND  ratio)  OR  (neutrophil-to-lymphocyte)  OR  NLR)

#2

ALL=(Coronary   )

#3

ALL= (Kawasaki)

#4

#1 AND #2 AND #3

Google scholar

N= 824

( ( neutrophil AND lymphocyte AND ratio ) OR ( neutrophil-to-lymphocyte ) OR NLR ) AND ( coronary ) AND (Kawasaki)

where my words occur

anywhere in the article

in the title of the article

Embase

N= 147

( ( neutrophil AND lymphocyte AND ratio ) OR ( neutrophil-to-lymphocyte ) OR NLR ) AND ( coronary ) AND (kawasaki)

ProQuest

N= 51

((neutrophil AND lymphocyte AND ratio) OR (neutrophil-to-lymphocyte) OR NLR) AND ab(Kawasaki) AND ab(Coronary)

TRIP database

N= 49

( ( neutrophil AND lymphocyte AND ratio ) OR ( neutrophil-to-lymphocyte ) OR NLR ) AND ( coronary ) AND (kawasaki)

Cochrane library

N= 0

( ( neutrophil AND lymphocyte AND ratio ) OR ( neutrophil-to-lymphocyte ) OR NLR ) AND ( coronary ) AND (kawasaki) in Title Abstract Keyword - (Word variations have been searched)

ScienceDirect

N= 93

( ( neutrophil AND lymphocyte AND ratio ) OR ( neutrophil-to-lymphocyte ) OR NLR ) AND ( coronary ) AND (kawasaki)
