# Supplementary material for: Neutrophil to Lymphocyte Ratio as a Biomarker for Predicting the Coronary Artery Abnormality in Kawasaki Disease: A Meta-Analysis
Source: Dis Markers. 2022 Oct 11;2022:6421543. doi: 10.1155/2022/6421543 (PMC9578863; doi:10.1155/2022/6421543)
Supplement: Supplementary Materials — The exact search strategy of each database is shown as supplementary file A. The list of included studies is shown in supplementary file B. [file 6421543.f1.zip › supplementary file B.rtf]

Title 
Neutrophil to lymphocyte ratio as a biomarker for predicting the coronary artery abnormality in Kawasaki disease: a meta-analysis
List of included studies

1. Bozlu G, Karpuz D, Hallioglu O, Unal S, Kuyucu N. Relationship between mean platelet volume-to-lymphocyte ratio and coronary artery abnormalities in Kawasaki disease. Cardiology in the Young 2018; 28 (6):832-836.

2. Chang LS, Lin YJ, Yan JH, Guo MM, Lo MH, Kuo HC. Neutrophil-to-lymphocyte ratio and scoring system for predicting coronary artery lesions of Kawasaki disease. BMC pediatrics 2020; 20 (1):398.

3. Chantasiriwan N, Silvilairat S, Makonkawkeyoon K, Pongprot Y, Sittiwangkul R. Predictors of intravenous immunoglobulin resistance and coronary artery aneurysm in patients with Kawasaki disease. Paediatrics and international child health 2018; 38 (3):209-212.

4. Cho HJ, Bak SY, Kim SY, Yoo R, Baek HS, Yang S, et al. High neutrophil : lymphocyte ratio is associated with refractory Kawasaki disease. Pediatrics international : official journal of the Japan Pediatric Society 2017; 59 (6):669-674.

5. Demir F, Karadeniz C, Özdemir R, Yozgat Y, Çelegen K, Karaaslan U, et al. Usefulness of Neutrophil to Lymphocyte Ratio in Prediction of Coronary Artery Lesions in Patients with Kawasaki Disease. Balkan medical journal 2015; 32 (4):371-376.

6. Gayretli Aydýn ZG, Tanýr G. Kawasaki Hastalýðýnda Koroner Arter Tulumunun Belirlenmesinde Lenfosit Monosit Oranýnýn Deðeri Kawasaki Hastalýðý: Lenfosit Monosit Oraný. Medical Journal of Bakirkoy 2019; 15 (2).

7. Ha KS, Jang GY, Lee J, Lee KC, Son CS. Laboratory Markers in Incomplete Kawasaki Disease according to Coronary Artery Outcome. Korean circulation journal 2018; 48 (4):287-295.


8. Ha KS, Lee J, Jang GY, Lee J, Lee KC, Son CS, et al. Value of neutrophil-lymphocyte ratio in predicting outcomes in Kawasaki disease. The American journal of cardiology 2015; 116 (2):301-306.

9. Haiyan G, Jianming L, Suqian T, Dong Q, Shuang L, Jin Z. Blood routine risk factors for coronary artery aneurysm in infants younger than 8 months with Kawasaki disease. BMC pediatrics 2022; 22 (1):1-8.

10. Hu J, Ren W. Analysis of Risk Factors of Kawasaki Disease With Coronary Artery Lesions. 2021.

11. Hua W, Ma F, Wang Y, Fu S, Wang W, Xie C, et al. A new scoring system to predict Kawasaki disease with coronary artery lesions. Clinical Rheumatology 2019; 38 (4):1099-1107.

12. Liu J, Huang Y, Chen C, Su D, Qin S, Pang Y. Risk Factors for Resistance to Intravenous Immunoglobulin Treatment and Coronary Artery Abnormalities in a Chinese Pediatric Population With Kawasaki Disease: A Retrospective Cohort Study. Frontiers in pediatrics 2022; 10.

13. Nakada T. Acute phase treatment for prevention of coronary artery stenosis caused in Kawasaki disease: a single center retrospective study. Journal of Advanced Research in Medicine 2018; 5 (4):1-7.

14. Yang M, Weng H, Pei Q, Jing F, Yi Q. The Relationship between Retinol-Binding Protein 4 and Markers of Inflammation and Thrombogenesis in Children with Kawasaki Disease. Mediators of inflammation 2021; 2021:7029514.

15. 윤±송Û이Ì, 주Ö희ñ영µ, 이Ì경æ석®, 차÷성º호£, 한Ñ미Ì영µ, 윤±경æ림². Severe Skin Lesions or Arthritis May be Associated with Coronary Artery Lesions in Kawasaki Disease. Pediatric Infection and Vaccine 2016; 23 (2):102-108.

16. 杨î燕à飞É, 张Å丽ö萍¼. 心S率¦变ä异ì性Ô指¸标ê和Í中Ð性Ô粒£细¸胞û与ë淋Ü巴Í细¸胞û比È值µ对Ô小¡儿ù川¨崎é病¡并¢发¢冠Ú状´动¯脉ö病¡变ä的Ä诊ï断Ï价Û值µ. 发¢育ý医½学§电ç子Ó杂Ó志¾ 2022; 10 (3):174-181.

17. 陈Â芃M螈¢, 杨î超¬, 李›刚Õ, 周ü超´然R. 系n统³性Ô免â疫ß-炎Š症Ç指w数”对Ô川¨崎é病¡患¼儿ù冠Ú脉ö扩©张Å的Ä预¤测â价Û值µ. 中†国‘免Æ疫u学w杂Ó志¾ 2020; 36 (16):2003.
